# The Commencement of Medical Study

**Published:** 1895-09-07

**Authors:** 


### The Commencement of Medical Study.

When the would-be student has decided that medicine is to be his career in life he ought at once to look many years ahead, and to enter upon certain questions regarding diplomas and degrees which his friends will probably urge him to put off till later in his course. It seems but natural to imagine that the first step is to become a student, and that all questions about qualifications can be decided when the time for qualifying draws nearer; but it is not so. Unless the student lays down his course correctly from the very beginning he is liable to great disappointments at the end. This arises partly from the course of study for a degree being somewhat, although not very, different from that required for a diploma, but it especially depends on the fact, which every student ought to recognise from the first, that the entrance examinations are not interchangeable, and that if a degree is wanted the appropriate entrance examination should be passed at the university at which the degree is to be taken.

The advantage of possessing a degree in medicine, so far as public estimation is concerned, is undoubted, and therefore it is desirable above all things to pass a preliminary which will admit to one or other of the degree giving bodies, viz., the Universities of Oxford, Cambridge, Durham, or London, the Victoria University, the Universities of Edinburgh, Aberdeen, Glasgow, St. Andrews, Dublin, and the Royal University of Ireland. We cannot here enter into the details of these various examinations, but we warn the student, so soon as he has decided on the degree that he desires, to obtain from headquarters exact details as to the examinations to be passed and the residence required. The medical degrees of any of the above universities are full qualifications for practice, and will enable their possessors to be placed upon the Medical Register.

To people of an ambitious turn of mind, however, it must be remembered that a degree, although a full qualification, will not enable a man to get on the staff of a London hospital either as a physician or surgeon, and the same applies to many hospitals out of London also. Anyone, therefore, who aims high, and hopes ultimately to obtain consulting rank, must become either a Member of the College of Physicians or a Fellow of the College of Surgeons. Fortunately the examinations of these two bodies have been so far amalgamated that passing the conjoint or qualifying examination paves the way to the higher ranks of each. The licensing bodies, other than the universities mentioned above, are—the conjoined Royal College of Physicians of London and Royal College of Surgeons of England; the conjoined Royal College of Physicians of Edinburgh, Royal College of Surgeons of Edinburgh, and Faculty of Physicians and Surgeons of Glasgow; the conjoined Royal College of Physicians of Ireland and Royal College of Surgeons in Ireland; and the Apothecaries' Society of London. It will be observed that while in times gone by each licensing body gave its own partial qualification independently, the different bodies have now been so grouped that no qualification can be obtained unless it is so complete as to give a registerable title to practice, and curiously it has turned out that the Apothecaries' Society of London is the only body except the universities which can single-handed give a complete licence to practice.

It is important to remember that, whereas the entrance examinations to the different universities are special to themselves, they all allow the student who has passed them to "register as a medical student," which is all that the non-university qualifying bodies require as a preliminary to their courses of study. It thus happens that by taking a university matriculation as one's entrance, one is free to go on to a degree if one likes, whereas if one takes any other entrance examination one may have to go back again to latin and mathematics if, later on, it should appear desirable to take a degree. We need have no hesitation in saying that, on the whole, the best course for a student who can afford the money and the time is to go first to Cambridge, taking the B A. degree and studying natural science, including anatomy and physiology; and then, after coming to London for his more purely medical work, taking his medical degree at Cambridge and also passing the London conjoint examination. But, for London students who aspire only to the Conjoint Diploma (which will leave them *Mr.* Jones or *Mr.* Smith, as the case may be, for the rest of their lives), the examination held by the College of Preceptors will probably be found the most convenient preliminary. Full particulars of the various recognised examinations may be obtained from the Registrar of the General Medical Council, 299, Oxford Street, W. We would, however, very strongly advise all London students to matriculate at the University of London, or in default of that at the University of Durham, so as to be able later on to gain a degree, the advantage of Durham over other extra-metropolitan universities being that only one year's residence is required.

The same applies to students at Birmingham, Bristol, and Sheffield as to those in London. Nothing can be better than some of these schools. At Birmingham, for example, the medical faculty of Mason College is a splendidly appointed centre of medical teaching, but like the London schools these colleges are affiliated to the London University alone, and only through it can obtain a degree for their students. At these colleges, therefore, the choice again lies between the London and the Durham Universities. In Leeds, Manchester, and Liverpool, however, we find three thoroughly equipped colleges, which together form the Victoria University. This university gives degrees which have quickly won for themselves a high repute, and all three colleges are models of their kind; the first duty of students entering at any one of them is then to matriculate at the Victoria University.

Durham with its medical faculty at Newcastle offers degrees to its own students, and after a year's residence to others also.

The Scotch students, again, are well off, for they all have universities at their own doors, and it is their own fault if they start practice without a degree, and the same may be said of the Irish.

The chief difficulty is as to the students in London, Birmingham, Sheffield, and Bristol, all of whom want ordinary degrees, and yet have to depend on the London University, which only gives what is practically an honours degree.

